# Sag deletion promotes DMBA/TPA‐induced skin carcinogenesis via YAP accumulation

**DOI:** 10.1002/mco2.648

**Published:** 2024-07-17

**Authors:** Yi Sun, Jie Xu, Dongping Wei, Hua Li

**Affiliations:** ^1^ Cancer Institute (Key Laboratory of Cancer Prevention and Intervention, China National Ministry of Education) of the Second Affiliated Hospital, and Institute of Translational Medicine, Zhejiang University School of Medicine Hangzhou China; ^2^ Research Center for Life Science and Human Health of Binjiang Institute Zhejiang University Hangzhou China; ^3^ Department of Radiation Oncology University of Michigan Ann Arbor Michigan USA

Dear Editor,

SAG (Sensitive to Apoptosis), also known as RBX2 (RING‐box protein‐2), is the RING subunit of Cullin‐RING ligase‐1 (CRL‐1) and CRL‐5, required for their ligase activities. Our recent studies found that in *Kras^G12D^*‐induced mouse tumor models, Sag played an oncogenic or tumor‐suppressive role in an organ‐dependent manner.^1^, ^2^, ^3^, ^4^, ^5^ Specifically, *Sag* deletion significantly inhibited lung tumorigenesis by causing the accumulation of a variety of tumor suppressor proteins, including NOXA, p21/p27, DEPTOR, and IκBα.^2^ In the pancreas, *Sag* deletion converted mPanINs to neoplastic cystic lesions by causing Shoc2 accumulation,^3^ whereas Sag transgenic expression promoted mPanIN1 formation as the early event, and impairs pancreatic functions as the late event by causing accumulation of Deptor and Nrf2.^5^ However, in the skin, *Sag* deletion driven by a leaky expression of Pdx1‐Cre promoted skin tumorigenesis through blocking autophagy and senescence by causing accumulation of Erbin to inactivate the Ras–Raf pathway, and of Nrf2 to scavenge the reactive oxygen species.^4^ Our earlier study also found that in a 7,12‐dimethylbenz[a]anthracene/phorbol ester 12‐O‐tetradecanoylphorbol 13‐acetate (DMBA/TPA)‐induced skin carcinogenesis model, transgenic SAG expression inhibits the early‐stage tumor progression by promoting c‐Jun degradation to inhibit AP‐1, and accelerates the late‐stage tumor growth by promoting IκBα degradation to activate NF‐κB and inhibit apoptosis.^1^ Whether and how *Sag* deletion affects skin carcinogenesis induced by DMBA/TPA is previously unknown.

In this study, we generated a *K5‐Cre;Sag^fl/fl^* (designated as *Sag^−/−^*) mouse model in FVB/N background, in which *Sag* is conditionally deleted in the skin, driven by K5‐Cre, along with the control mice with genotype of *Sag^fl/fl^* (designated as *Sag^+/+^*). Mice at the age of 7−8 weeks were used for the DMBA/TPA two‐stage carcinogenesis protocol. Specifically, a single dose of DMBA (100 nmol) was topically applied to the shaved backs of mice, and TPA (5 nmol) was, 2 weeks after initiation, administrated twice weekly to the dorsal skin for 20 weeks. While *Sag^+/+^* control mice developed a very limited number of skin tumors only in two out of seven mice, *Sag^−/‐^* mice developed a much greater number of tumors in 4 out of 5 mice (Figure 1A, top and middle panels, and Figure S1A), indicating that *Sag* deletion promoted the development of skin tumors in this two‐stage chemical carcinogenesis model.

**FIGURE 1:**
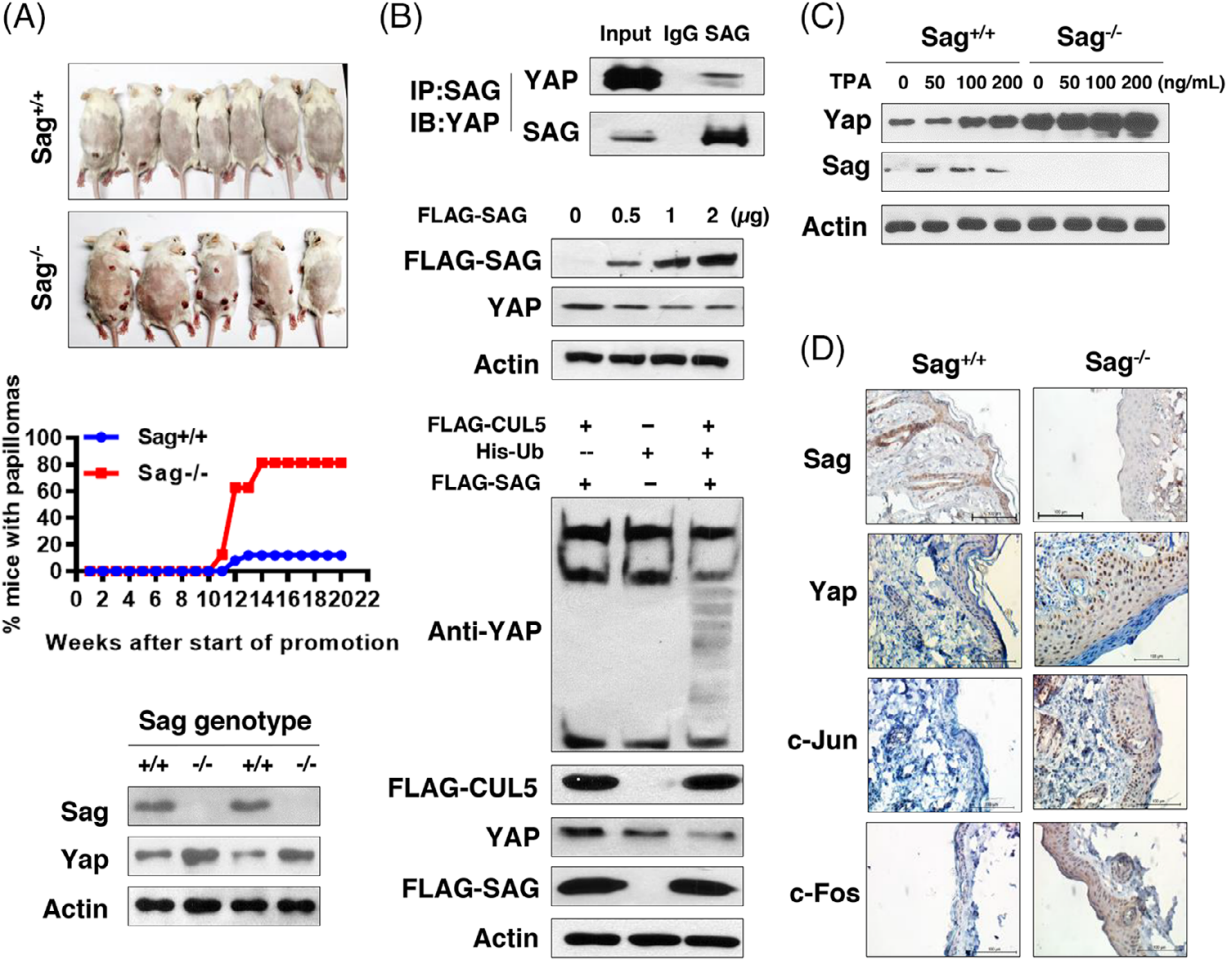
Sag deletion promotes skin tumorigenesis induced by DMBA‐TPA by causing YAP accumulation. (A) Sag deletion promotes skin tumorigenesis induced by DMBA‐TPA: Visualization of mouse skin tumors with *Sag^+/+^* and *Sag^−/−^* genotypes (top panel); Time course of skin tumor development in *Sag^+/+^* and *Sag^−/−^* mice (middle panel); and Western blotting analysis of primary keratinocytes from *Sag^+/+^* and *Sag^−/−^* skin tissues, using indicated Abs (bottom panel). (B) YAP is a new substrate of Sensitive to Apoptosis (SAG)‐CUL5 E3 ligase: The interaction between SAG and YAP under physiological conditions. The lysates from HEK 293 (Human epithelial kidney 293) cells were immune‐precipitated with anti‐SAG Ab, along with IgG control, followed by IB with YAP Ab (top panel): Ectopic SAG expression reduces YAP levels in a dose‐dependent manner: UMSCC11B skin tumor cells were transfected with increasing amount of plasmid encoding FLAG‐SAG, followed by IB with indicated Abs (middle panel); SAG‐CUL‐5 promotes YAP polyubiquitylation. The HEK 293 cells were transfected with indicated plasmids, followed by an in vivo polyubiquitylation assay (bottom panel). (C) YAP induction by TPA in keratinocytes: Primary keratinocytes were established from mouse skin of *Sag^+/+^* and *Sag^−/−^* mice, and subjected to TPA treatment with indicated concentrations for 3 h, followed by IB with indicated Abs. (D) Sag deletion causes accumulation of Yap, c‐Jun, and c‐Fos in mouse skin: immunohistochemistry (IHC) staining of skin tissues from *Sag^+/+^* and *Sag^−/−^* mice after TPA treatment for 4 weeks, using indicated Abs.

To pursue the underlying mechanism, we hypothesized that *Sag* deletion would cause the accumulation of the oncogenic proteins to promote carcinogenesis. We then focused on yes‐associated protein (YAP), one oncogenic protein in the Hippo pathway, shown to be accumulated in pancreatic tissues upon *Sag* deletion during *Kras^G12D^*‐induced tumorigenesis.^3^ The Western blotting of two pairs of primary keratinocytes derived from the skin of *Sag^+/+^* and *Sag^−/‐^* mice showed expected *Sag* deletion, as well as Yap accumulation in *Sag^−/‐^* keratinocytes (Figure 1A, bottom panel).

We next determined whether YAP is indeed a new substrate of SAG E3 ligase. The immunoprecipitation‐based pulldown assay showed that endogenous SAG binds to endogenous YAP (Figure 1B, top panel). Ectopic expression of SAG reduced endogenous levels of YAP in a dose‐dependent manner in UMSCC11B head/neck carcinoma cells (Figure 1B, middle panel), as well as in SK‐BR3 breast cancer cells and H1299 lung cancer cells (Figure S1B). Furthermore, SAG in combination with cullin‐5 (CUL‐5) promotes the polyubiquitylation of YAP (Figure 1B, bottom panel).

Finally, we found that the primary keratinocytes established from *Sag^−/‐^* skin had much higher basal levels of YAP than that from *Sag^+/+^* skin. Furthermore, YAP is subject to TPA upregulation, as evidenced by YAP induction by TPA treatment in a dose‐dependent manner (Figure 1C), which is likely due to the AP‐1 transactivation induced by TPA via the AP‐1 binding sites found in the promoter sequences of both human and mouse YAP genes with one mismatch (Figure S1C). The results of immunohistochemistry staining showed that in mouse skin tissues treated with 4 weeks of TPA treatment, Sag is expectedly deleted in the *Sag^−/‐^* skin, and YAP staining was significantly increased, along with the increased staining of c‐Jun and c‐Fos as the positive controls in the skin,^1^ as compared to the *Sag^+/+^* skin (Figure 1D). Furthermore, in the *Sag^−/‐^* skin, the TPA treatment also increased skin thickness with positive staining for Ki67 (proliferation) and CK19 (malignancy) (Figure S1D). Taken together, our study demonstrated that YAP is a new substrate of SAG‐CUL5 E3 ligase in the skin, and *Sag* deletion, by causing YAP accumulation, promotes skin proliferation by TPA, and skin carcinogenesis by DMBA/TPA.

In summary, our study fits the following working model: during skin carcinogenesis induced by DMBA‐TPA and skin proliferation induced by TPA, Sag couples with Cul‐5 to promote ubiquitylation and degradation of oncogenic proteins (e.g., Yap and c‐Jun), leading to suppression of tumor progression. Upon *Sag* deletion, this growth‐suppressive effect was abrogated, resulting in accelerated proliferation and tumor progression. Thus, Sag appears to be a tumor suppressor in the skin.

## AUTHOR CONTRIBUTIONS

J.X. D.W. and H.L. performed experiments. J.X. and Y.S. analyzed data. Y.S. conceived, supervised the project, and wrote the manuscript. All authors have read and approved the final manuscript.

## CONFLICT OF INTEREST STATEMENT

The authors declare no conflict of interest.

## FUNDING INFORMATION

This project was supported by the National Key R&D Program of China (2021YFA1101000 to Y. S.) and the National Natural Science Foundation of China (U22A20317 and 92253203 to Y. S.).

## ETHICS STATEMENT

All procedures were approved by the University of Michigan Committee on Use and Care of Animals (PRO00008986). Animal care was provided in accordance with the principles and procedures outlined in the National Research Council Guide for the Care and Use of Laboratory Animals.

## Supporting information

Supporting Information

## Data Availability

The data are available from the corresponding author upon reasonable request.
