# Supplementary material for: Sag deletion promotes DMBA/TPA‐induced skin carcinogenesis via YAP accumulation
Source: MedComm (2020). 2024 Jul 17;5(8):e648. doi: 10.1002/mco2.648 (PMC11253290; doi:10.1002/mco2.648)
Supplement: Supplementary file 1 — Supporting Information [file MCO2-5-e648-s001.docx]

**Sag deletion promotes DMBA/TPA-induced skin carcinogenesis**

**via YAP accumulation**

Yi Sun^1,2,^*, Jie Xu^3^, Dongping Wei^3^, and Hua Li^3^

^1^Cancer Institute (Key Laboratory of Cancer Prevention and Intervention, China National Ministry of Education) of the Second Affiliated Hospital, and Institute of Translational Medicine, Zhejiang University School of Medicine, Hangzhou 310029, China.

^2^Research Center for Life Science and Human Health of Binjiang Institute, Zhejiang University, Hangzhou 310053, China.

^3^ Department of Radiation Oncology, University of Michigan, Ann Arbor, MI 48109, USA

* To whom correspondence should be addressed: Yi Sun, [yisun@zju.edu.cn](mailto:yisun@zju.edu.cn)

**Material and methods**

1. Mice

The Sag^fl/fl^ mouse model were established as described previously ^1^ and backcrossed into FVB/N background for 6 generations. K5-Cre mice in FVB/N background were a gift from Dr. A. Dlugosz from University of Michigan. The K5-Cre;Sag^fl/fl^ strain were generated by two rounds of crossing between K5-Cre and Sag^fl/fl^ mice.

2. Establishment of primary keratinocytes from mouse skin.

Primary keratinocytes were isolated from dorsal skin of pups at p1–2 as described previously ^1^. Brief, newborn mice (1–2 d old) were sacrificed by CO_2_ and washed with 70% ethanol. Skin was cut on the dorsal side along the length of the body. The isolated skin was stretched, with dermal side down, on a 0.25% trypsin surface in a culture dish and incubated overnight at 4°C. The next day, the dermis was separated from the epidermis and suspended in high calcium minimum essential medium Eagle (EMEM; supplemented with 8% FBS, 1.4 mM CaCl_2_, 1 ng/ml keratinocyte growth factor, 1,000 U/ml penicillin, and 1,000 µg/ml streptomycin). The cell suspension was centrifuged at 150 g for 5 min at 4°C, re-suspended in high calcium EMEM, and filtered through a 70-µm cell strainer (BD) into a new 50-ml conical tube. Cells were centrifuged at 150 g for 5 min at 4°C and resuspended in low calcium EMEM (supplemented with 8% FBS, 0.05 mM CaCl_2_, 1 ng/ml keratinocyte growth factor, 100 U/ml penicillin, and 100 µg/ml streptomycin) for cell culture.

3. The *in vivo* ubiquitylation assay.

The assay was performed as described ^2^. Briefly, the 293 cells were cotransfected with FLAG-CUL-5, FLAG-SAG and His-Ub. Thirty-six hrs post-transfection, cells were lysed in 6 M guanidinium denaturing solution. All ubiquitylated proteins were purified by Ni-bead pull-down, and polyubiquitylated YAP was detected by IB using anti-YAP antibody.

4. H&E staining and immuno-histochemical staining

Mice at the end of TPA application were sacrificed and skin tumors were harvested, fixed in 10% formalin, embedded in paraffin and sectioned (4 μm), followed by H&E staining and examination under a microscope. For immunohistochemistry, the sections were deparaffinized in xylene and rehydrated through graded ethanol, antigen retrieval was performed for 20 min at 95 °C with 0.1% sodium citrate buffer (pH 6.0). Following quenching of endogenous peroxidase activity with 3% H_2_O_2_·dH_2_O and blocking of non-specific binding with 1% bovine serum albumin buffer, sections were incubated overnight at 4 °C with indicated antibody. Following several washes, the sections were treated with HRP conjugated secondary antibody for 30 min at room temperature, and stained with 0.05% 3, 3-diaminobenzidine tetrahydrochloride (DAB). The antibodies used are Ki67 (BD Bioscience, Cat. 550609), CK19 (Abcam, ab87000), c-JUN (Cell Signaling, #9165), FLAG (Sigma, Cat#F1804), SAG (clone: Sag-10)^1^, YAP (Cell Signaling Cat#14074), and actin (Sigma Cat#A-5316).

**References：**

**1.** Xie CM, Wei D, Zhao L, et al. Erbin is a novel substrate of the Sag-betaTrCP E3 ligase that regulates KrasG12D-induced skin tumorigenesis. *J Cell Biol.* Jun 8 2015;209(5):721-737.

**2.** Tan M, Chang Y, Liu X, et al. The Sag-Shoc2 axis regulates conversion of mPanINs to cystic lesions in Kras pancreatic tumor model. *Cell Rep.* Dec 20 2022;41(12):111837.

**
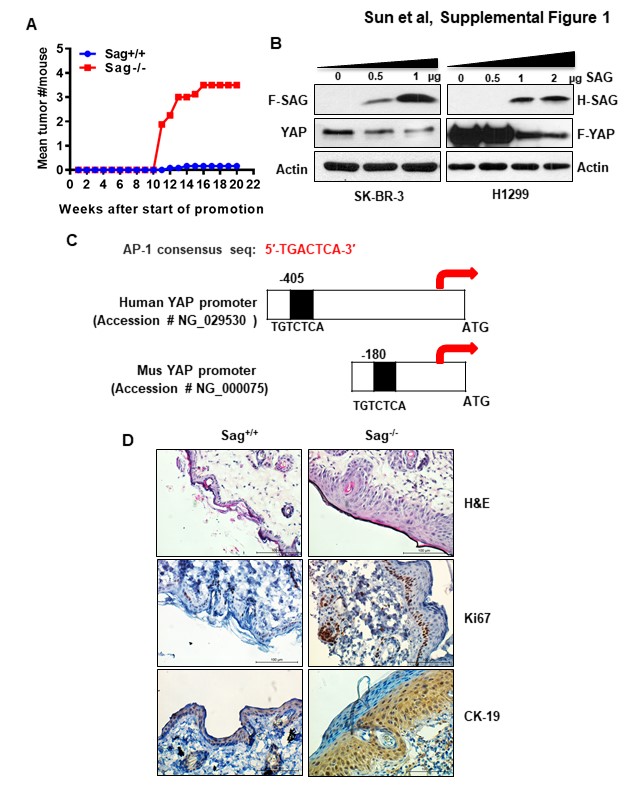
**

**Legends for supplemental figure.**

Figure S1. SAG regulates skin carcinogenesis via targeting YAP.

1. The time course of skin tumor development induced by DMBA-TPA. Shown is mean tumor # per mouse.
2. SAG reduced YAP level in a dose dependent manner. SK-BR3 cells were transfected with increasing amount of plasmid encoding FLAG-SAG (F-SAG), or H1299 cells were co-transfected with increasing amount of plasmids encoding HA-SAG (H-SAG) and FLAG-YAP (F-YAP), followed by Western blotting with indicated Abs.
3. Graphic plots of the promoter fragments of both human and mouse YAP genes with indicated locations of the AP1 binding sites upstream translational initiation site ATG.
4. TPA promotes skin proliferation in Sag^-/-^ mice. The dorsal skins of Sag^+/+^ and Sag^-/-^ mice were applied with TPA twice a week for 4 weeks, the skin tissues were harvested for H&E or IHC staining, using indicated Abs.
